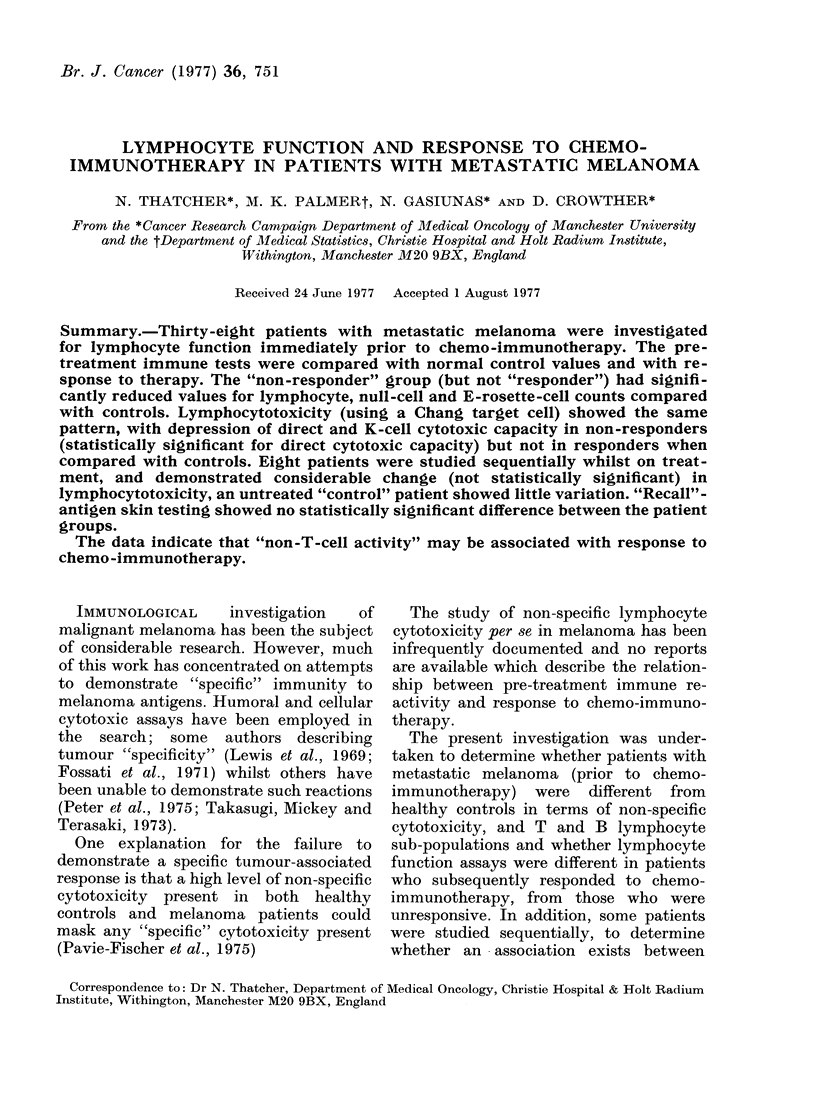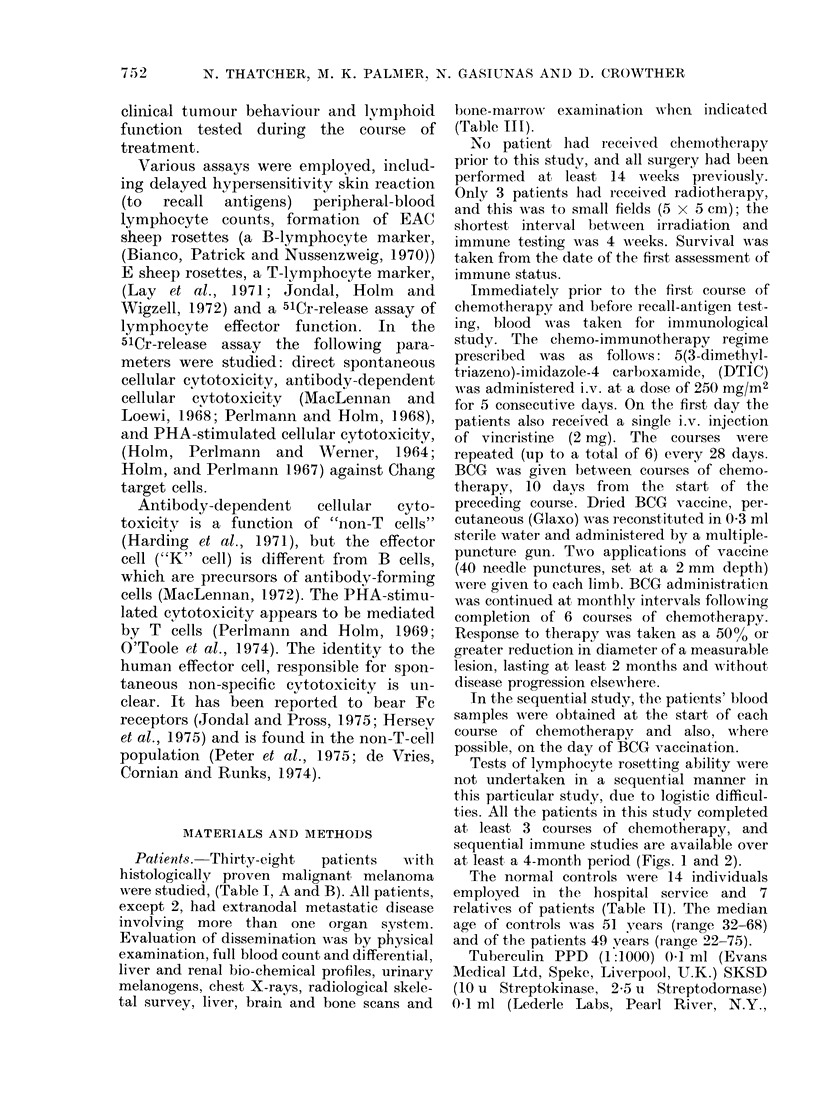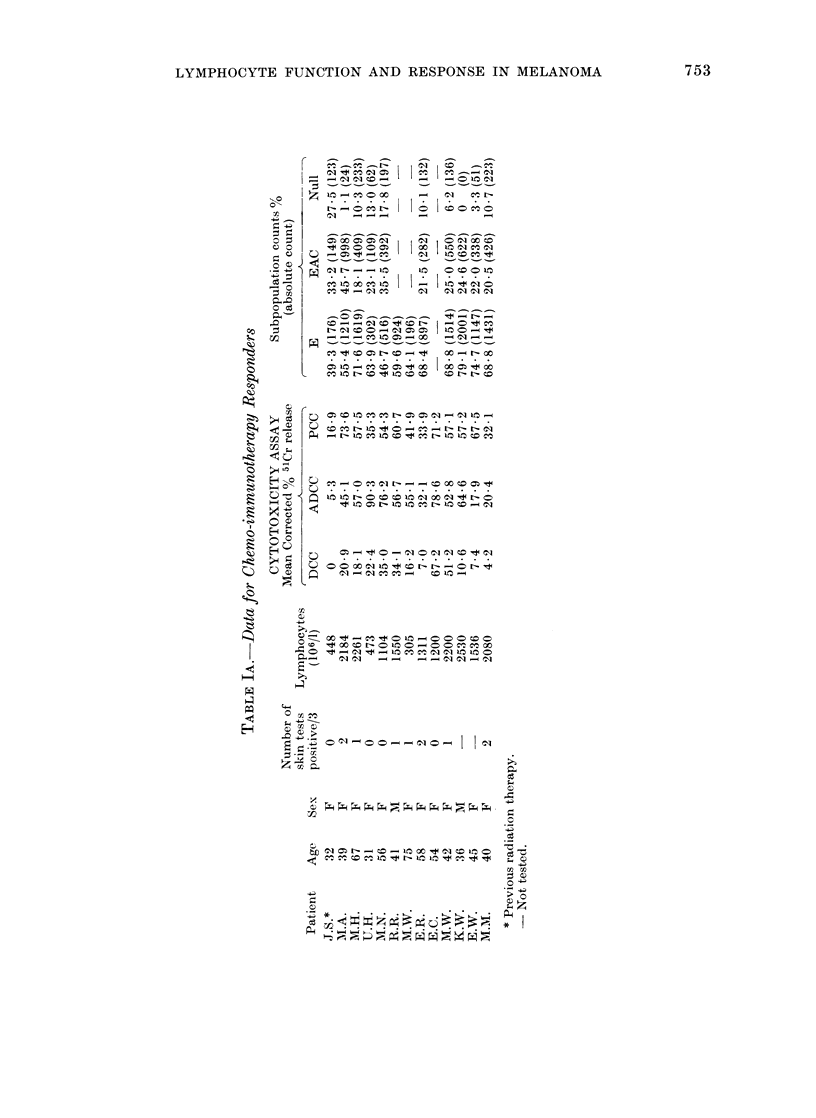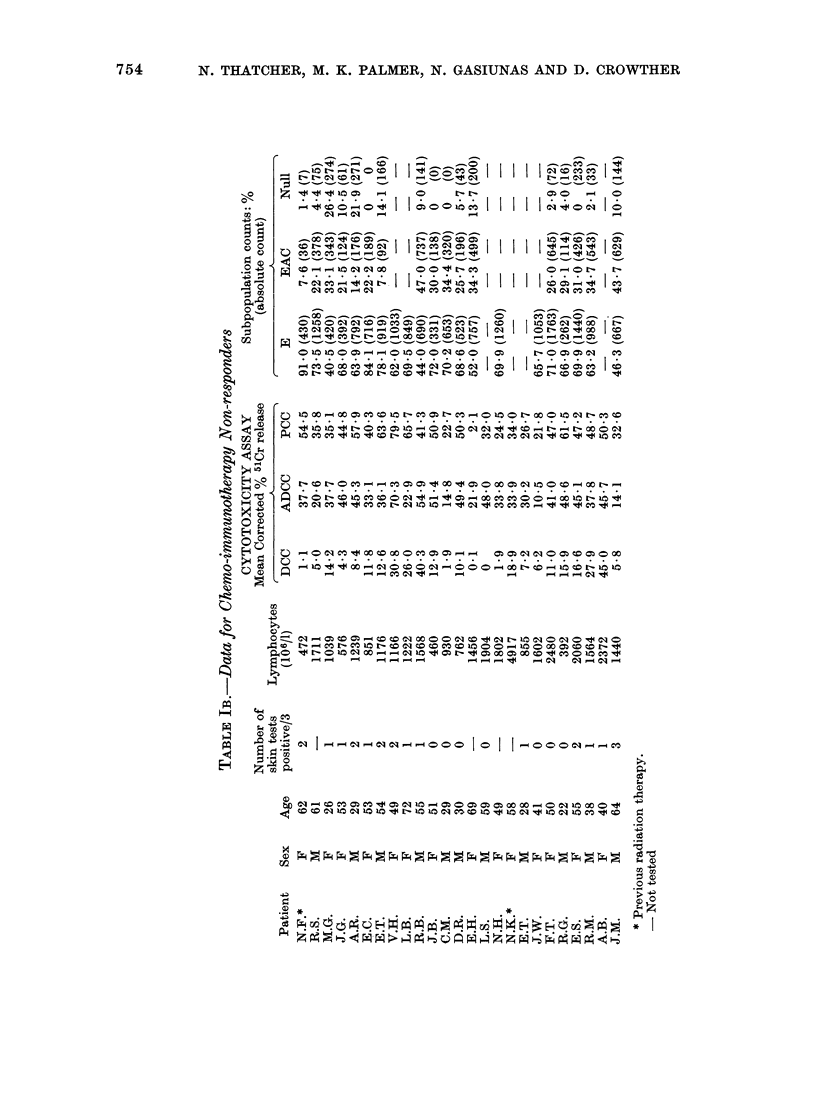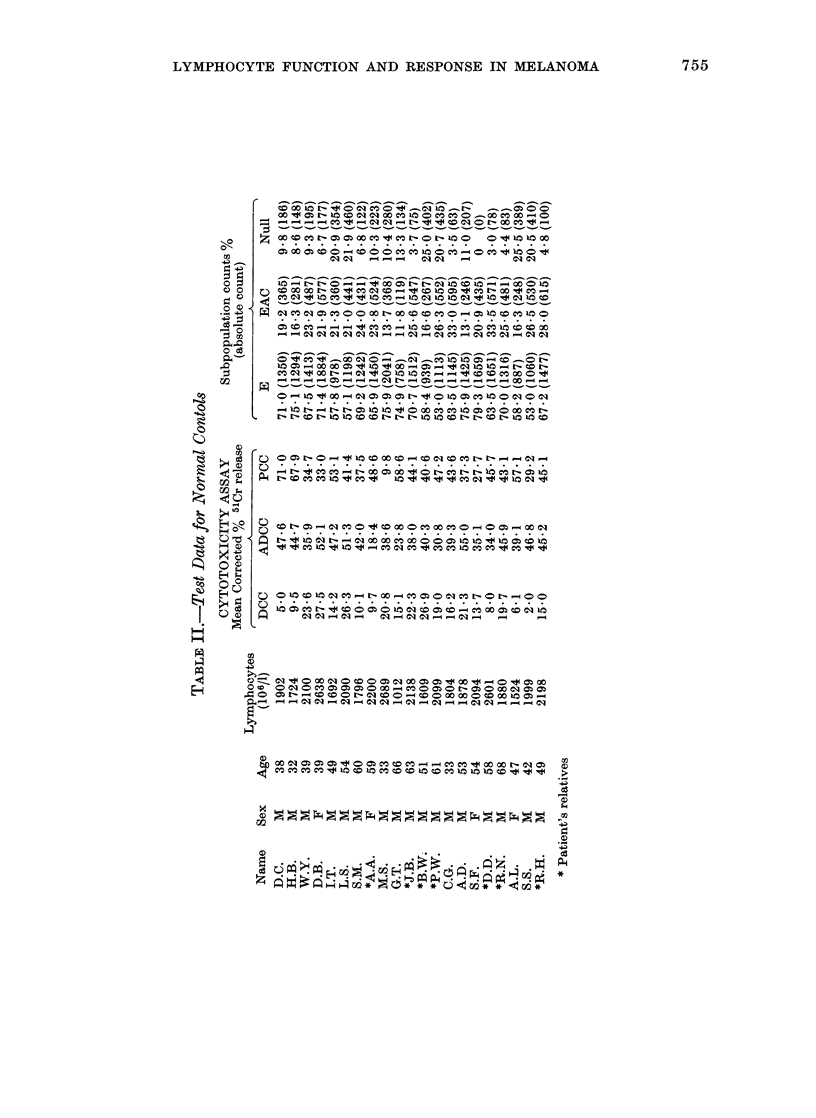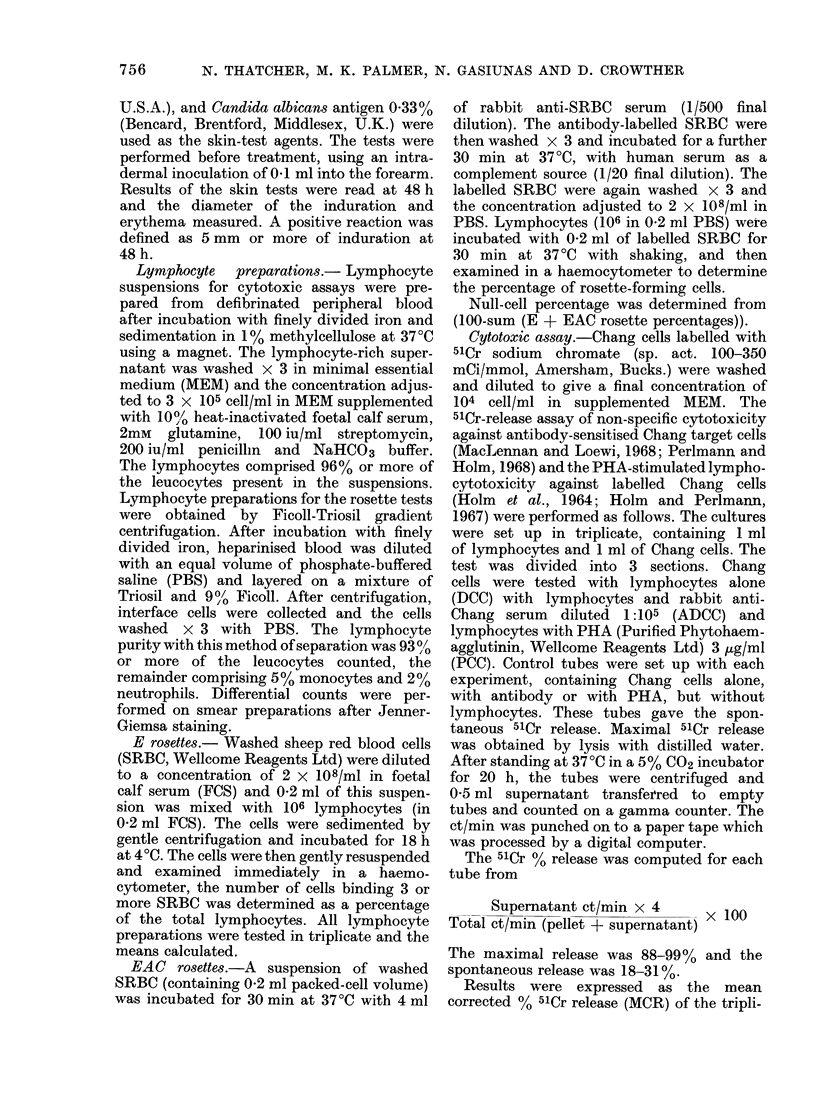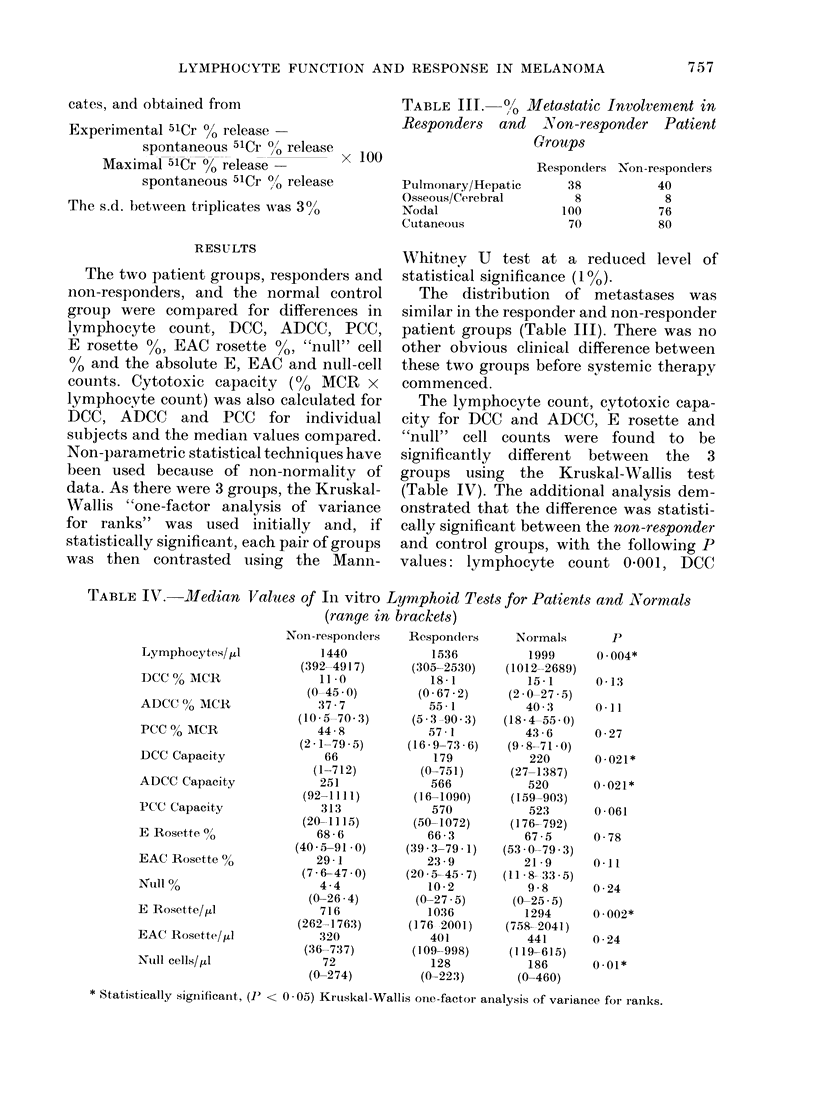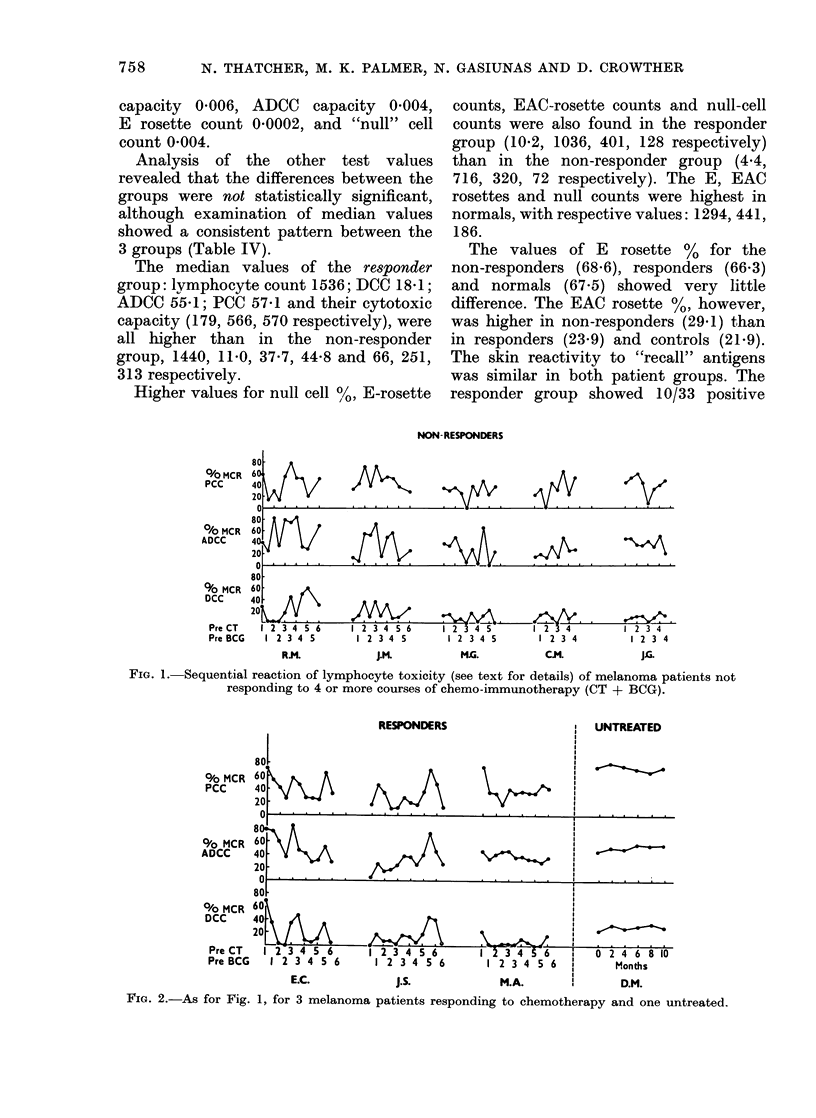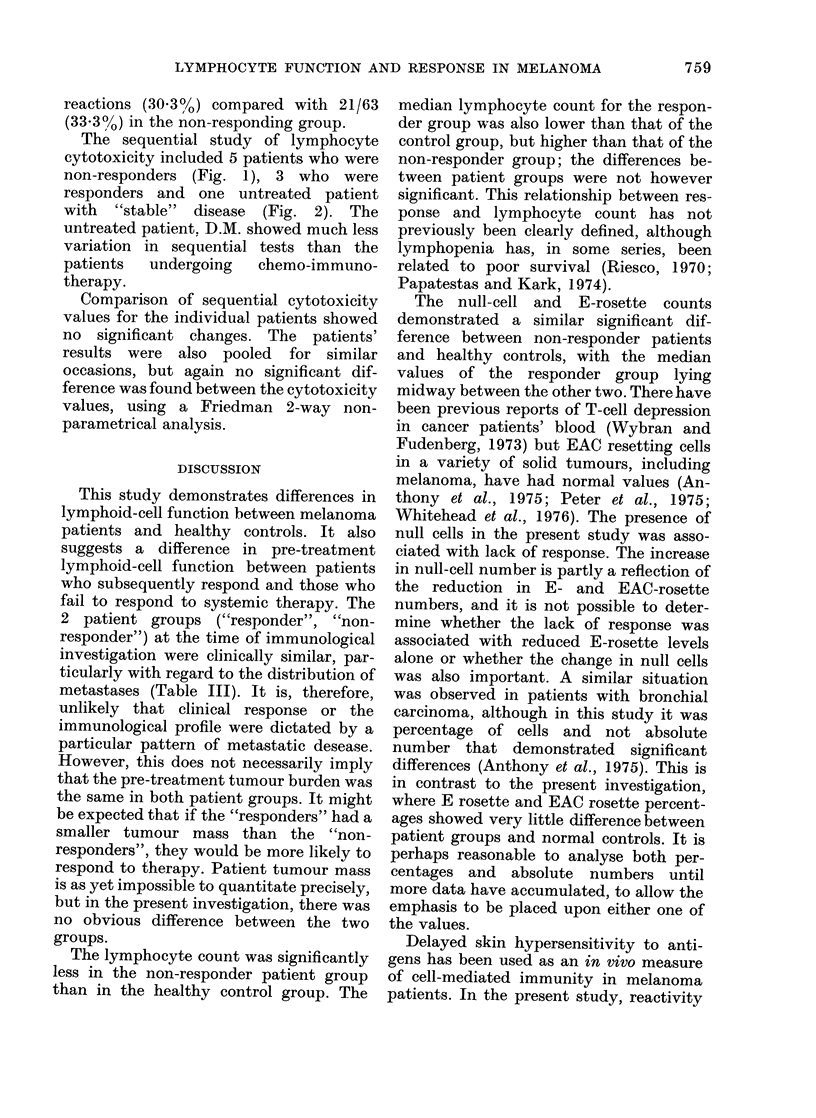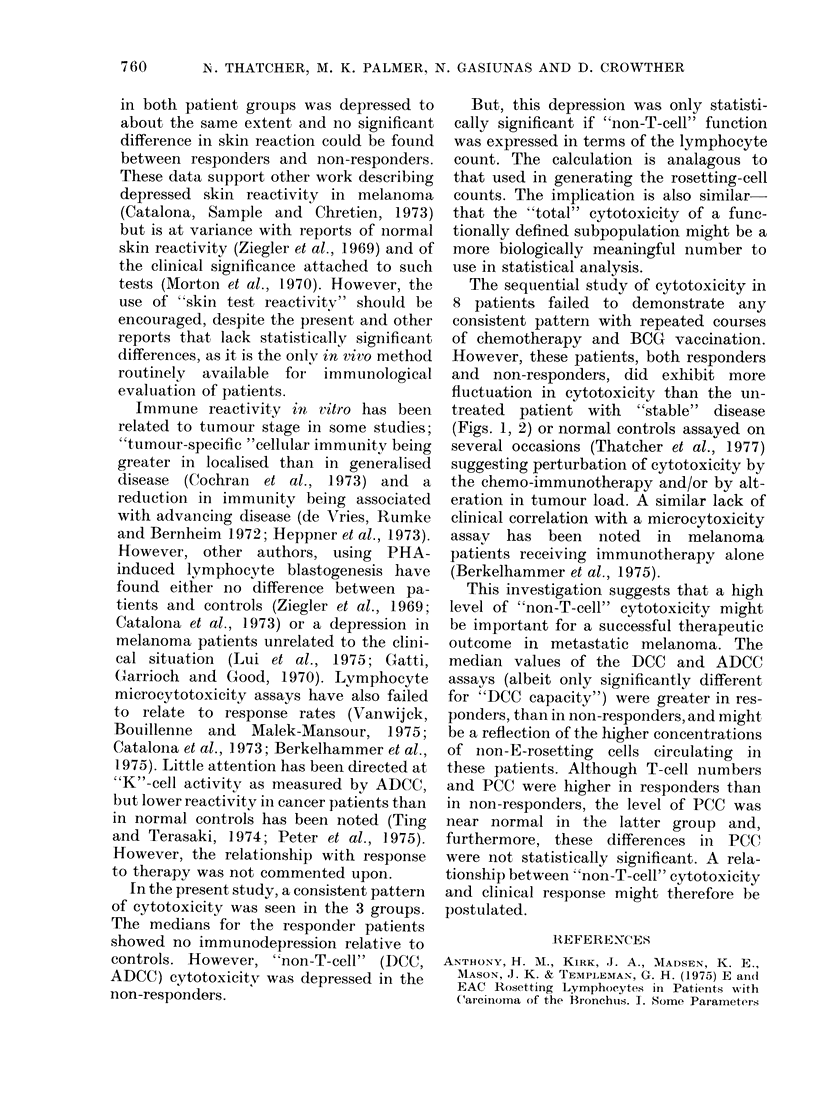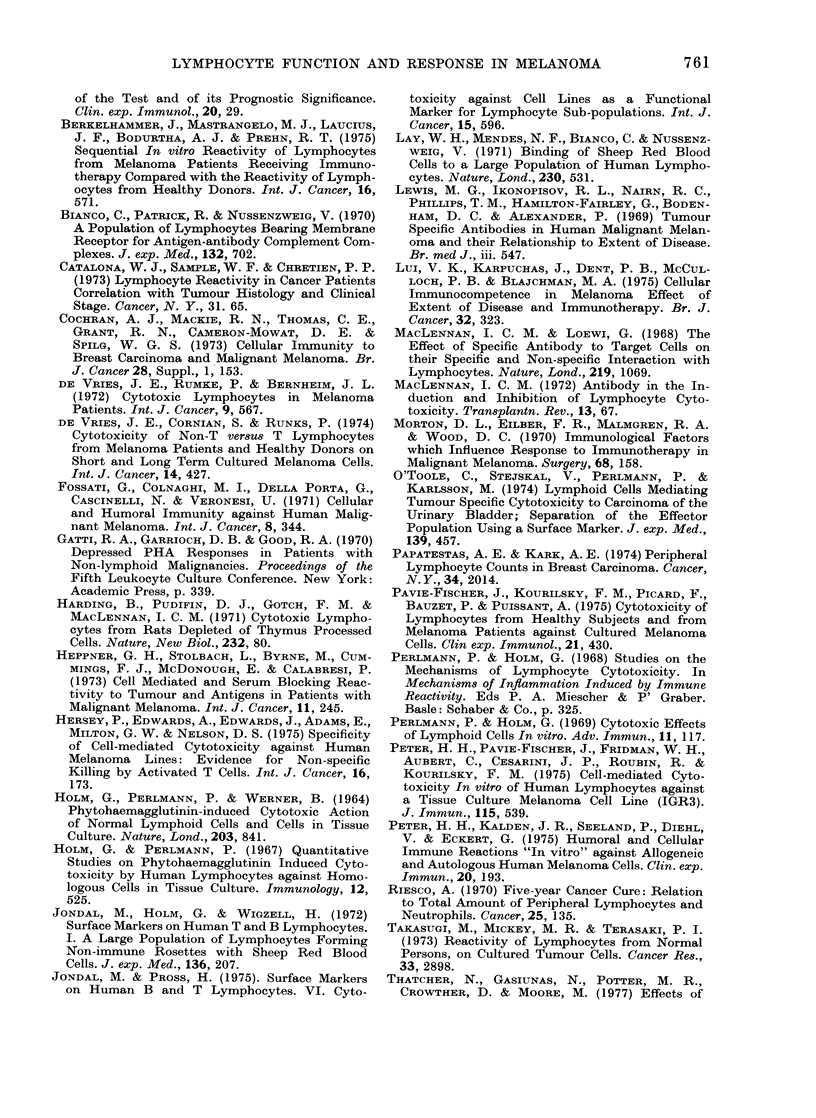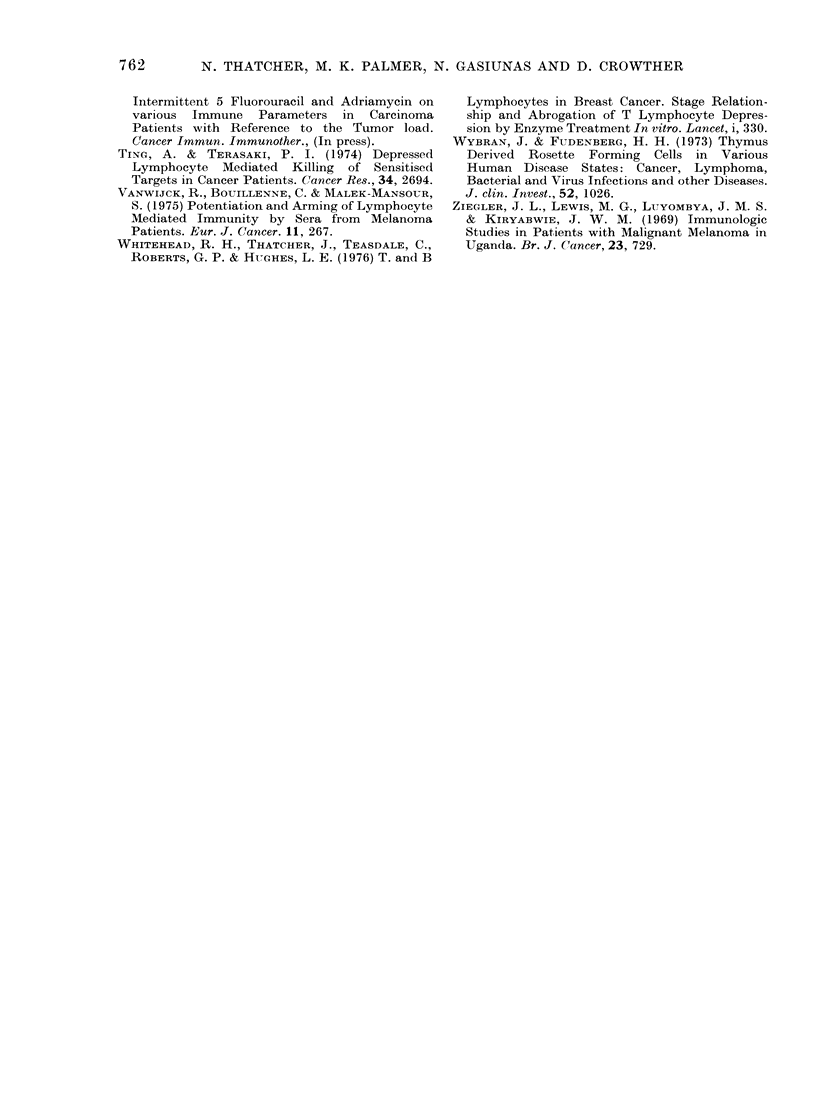# Lymphocyte function and response to chemo-immunotherapy in patients with metastatic melanoma.

**DOI:** 10.1038/bjc.1977.258

**Published:** 1977-12

**Authors:** N. Thatcher, M. K. Palmer, N. Gasiunas, D. Crowther

## Abstract

Thirty-eight patients with metastatic melanoma were investigated for lymphocyte function immediately prior to chemo-immunotherapy. The pre-treatment immune tests were compared with normal control values and with response to therapy. The "non-responder" group (but not "responder") had significantly reduced values for lymphocyte, null-cell and E-rosette-cell counts compared with controls. Lymphocytoxicity ( using a Chang target cell) showed the same pattern, with depression of direct and K-cell cytotoxic capacity in non-responders compared with controls. Eight patients were studied sequentially whilst on treatment, and demonstrated considerable change (not statistically significant) in lymphocytotoxicity, an untreated "control" patient showed little variation. "Recall"-antigen skin testing showed no statistically significant difference between the patient groups. The data indicate that "non-T-cell activity" may be associated with response to chemo-immunotherapy.


Br. J. Cancer (1977) 36, 751

### LYMPHOCYTE FUNCTION AND RESPONSE TO CHEMO-IMMUNOTHERAPY IN PATIENTS WITH METASTATIC MELANOMA

N. THATCHER*, M. K. PALMER†, N. GASIUNAS* AND D. CROWTHER*

*From the *Cancer Research Campaign Department of Medical Oncology of Manchester University and the †Department of Medical Statistics, Christie Hospital and Holt Radium Institute, Withington, Manchester M20 9BX, England*

Received 24 June 1977 Accepted 1 August 1977

**Summary.—Thirty-eight patients with metastatic melanoma were investigated for lymphocyte function immediately prior to chemo-immunotherapy. The pre-treatment immune tests were compared with normal control values and with response to therapy. The "non-responder" group (but not "responder") had significantly reduced values for lymphocyte, null-cell and E-rosette-cell counts compared with controls. Lymphocytotoxicity (using a Chang target cell) showed the same pattern, with depression of direct and K-cell cytotoxic capacity in non-responders (statistically significant for direct cytotoxic capacity) but not in responders when compared with controls. Eight patients were studied sequentially whilst on treatment, and demonstrated considerable change (not statistically significant) in lymphocytotoxicity, an untreated "control" patient showed little variation. "Recall"-antigen skin testing showed no statistically significant difference between the patient groups.**

**The data indicate that "non-T-cell activity" may be associated with response to chemo-immunotherapy.**

IMMUNOLOGICAL investigation of malignant melanoma has been the subject of considerable research. However, much of this work has concentrated on attempts to demonstrate "specific" immunity to melanoma antigens. Humoral and cellular cytotoxic assays have been employed in the search; some authors describing tumour "specificity" (Lewis *et al.*, 1969; Fossati *et al.*, 1971) whilst others have been unable to demonstrate such reactions (Peter *et al.*, 1975; Takasugi, Mickey and Terasaki, 1973).

One explanation for the failure to demonstrate a specific tumour-associated response is that a high level of non-specific cytotoxicity present in both healthy controls and melanoma patients could mask any "specific" cytotoxicity present (Pavie-Fischer *et al.*, 1975)

The study of non-specific lymphocyte cytotoxicity *per se* in melanoma has been infrequently documented and no reports are available which describe the relationship between pre-treatment immune reactivity and response to chemo-immunotherapy.

The present investigation was undertaken to determine whether patients with metastatic melanoma (prior to chemo-immunotherapy) were different from healthy controls in terms of non-specific cytotoxicity, and T and B lymphocyte sub-populations and whether lymphocyte function assays were different in patients who subsequently responded to chemo-immunotherapy, from those who were unresponsive. In addition, some patients were studied sequentially, to determine whether an association exists between

Correspondence to: Dr N. Thatcher, Department of Medical Oncology, Christie Hospital & Holt Radium Institute, Withington, Manchester M20 9BX, England clinical tumour behaviour and lymphoid function tested during the course of treatment.

Various assays were employed, including delayed hypersensitivity skin reaction (to recall antigens) peripheral-blood lymphocyte counts, formation of EAC sheep rosettes (a B-lymphocyte marker, (Bianco, Patrick and Nussenzweig, 1970)) E sheep rosettes, a T-lymphocyte marker, (Lay *et al.*, 1971; Jondal, Holm and Wigzell, 1972) and a $^{51}$Cr-release assay of lymphocyte effector function. In the $^{51}$Cr-release assay the following parameters were studied: direct spontaneous cellular cytotoxicity, antibody-dependent cellular cytotoxicity (MacLennan and Loewi, 1968; Perlmann and Holm, 1968), and PHA-stimulated cellular cytotoxicity, (Holm, Perlmann and Werner, 1964; Holm, and Perlmann 1967) against Chang target cells.

Antibody-dependent cellular cytotoxicity is a function of "non-T cells" (Harding *et al.*, 1971), but the effector cell ("K" cell) is different from B cells, which are precursors of antibody-forming cells (MacLennan, 1972). The PHA-stimulated cytotoxicity appears to be mediated by T cells (Perlmann and Holm, 1969; O'Toole *et al.*, 1974). The identity to the human effector cell, responsible for spontaneous non-specific cytotoxicity is unclear. It has been reported to bear Fc receptors (Jondal and Pross, 1975; Hersey *et al.*, 1975) and is found in the non-T-cell population (Peter *et al.*, 1975; de Vries, Cornian and Runks, 1974).

### MATERIALS AND METHODS

*Patients.*—Thirty-eight patients with histologically proven malignant melanoma were studied, (Table I, A and B). All patients, except 2, had extranodal metastatic disease involving more than one organ system. Evaluation of dissemination was by physical examination, full blood count and differential, liver and renal bio-chemical profiles, urinary melanogens, chest X-rays, radiological skeletal survey, liver, brain and bone scans and bone-marrow examination when indicated (Table III).

No patient had received chemotherapy prior to this study, and all surgery had been performed at least 14 weeks previously. Only 3 patients had received radiotherapy, and this was to small fields (5 × 5 cm); the shortest interval between irradiation and immune testing was 4 weeks. Survival was taken from the date of the first assessment of immune status.

Immediately prior to the first course of chemotherapy and before recall-antigen testing, blood was taken for immunological study. The chemo-immunotherapy regime prescribed was as follows: 5(3-dimethyl-triazeno)-imidazole-4 carboxamide, (DTIC) was administered i.v. at a dose of 250 mg/m$^2$ for 5 consecutive days. On the first day the patients also received a single i.v. injection of vincristine (2 mg). The courses were repeated (up to a total of 6) every 28 days. BCG was given between courses of chemotherapy, 10 days from the start of the preceding course. Dried BCG vaccine, percutaneous (Glaxo) was reconstituted in 0·3 ml sterile water and administered by a multiple-puncture gun. Two applications of vaccine (40 needle punctures, set at a 2 mm depth) were given to each limb. BCG administration was continued at monthly intervals following completion of 6 courses of chemotherapy. Response to therapy was taken as a 50% or greater reduction in diameter of a measurable lesion, lasting at least 2 months and without disease progression elsewhere.

In the sequential study, the patients' blood samples were obtained at the start of each course of chemotherapy and also, where possible, on the day of BCG vaccination.

Tests of lymphocyte rosetting ability were not undertaken in a sequential manner in this particular study, due to logistic difficulties. All the patients in this study completed at least 3 courses of chemotherapy, and sequential immune studies are available over at least a 4-month period (Figs. 1 and 2).

The normal controls were 14 individuals employed in the hospital service and 7 relatives of patients (Table II). The median age of controls was 51 years (range 32–68) and of the patients 49 years (range 22–75).

Tuberculin PPD (1:1000) 0·1 ml (Evans Medical Ltd, Speke, Liverpool, U.K.) SKSD (10 u Streptokinase, 2·5 u Streptodornase) 0·1 ml (Lederle Labs, Pearl River, N.Y.,

TABLE IA.—*Data for Chemo-immunotherapy Responders*

| Patient | Age | Sex | Number of skin tests positive/3 | Lymphocytes ($10^6$/l) | CYTOTOXICITY ASSAY Mean Corrected % $^{51}Cr$ release | | | Subpopulation counts % (absolute count) | | |
|---|---|---|---|---|---|---|---|---|---|---|
| | | | | | DCC | ADCC | PCC | E | EAC | Null |
| J.S.* | 32 | F | 0 | 448 | 0 | 5·3 | 16·9 | 39·3 (176) | 33·2 (149) | 27·5 (123) |
| M.A. | 39 | F | 2 | 2184 | 20·9 | 45·1 | 73·6 | 55·4 (1210) | 45·7 (998) | 1·1 (24) |
| M.H. | 67 | F | 1 | 2261 | 18·1 | 57·0 | 57·5 | 71·6 (1619) | 18·1 (409) | 10·3 (233) |
| U.H. | 31 | F | 0 | 473 | 22·4 | 90·3 | 35·3 | 63·9 (302) | 23·1 (109) | 13·0 (62) |
| M.N. | 56 | F | 0 | 1104 | 35·0 | 76·2 | 54·3 | 46·7 (516) | 35·5 (392) | 17·8 (197) |
| R.R. | 41 | M | 1 | 1550 | 34·1 | 56·7 | 60·7 | 59·6 (924) | — — | — — |
| M.W. | 75 | F | 1 | 305 | 16·2 | 55·1 | 41·9 | 64·1 (196) | — — | — — |
| E.R. | 58 | F | 2 | 1311 | 7·0 | 32·1 | 33·9 | 68·4 (897) | 21·5 (282) | 10·1 (132) |
| E.C. | 54 | F | 0 | 1200 | 67·2 | 78·6 | 71·2 | — — | — — | — — |
| M.W. | 42 | F | 1 | 2200 | 51·2 | 52·8 | 57·1 | 68·8 (1514) | 25·0 (550) | 6·2 (136) |
| K.W. | 36 | M | — | 2530 | 10·6 | 64·6 | 57·2 | 79·1 (2001) | 24·6 (622) | 0 (0) |
| E.W. | 45 | F | — | 1536 | 7·4 | 17·9 | 67·5 | 74·7 (1147) | 22·0 (338) | 3·3 (51) |
| M.M. | 40 | F | 2 | 2080 | 4·2 | 20·4 | 32·1 | 68·8 (1431) | 20·5 (426) | 10·7 (223) |

* Previous radiation therapy.

— Not tested.

TABLE IB.—*Data for Chemo-immunotherapy Non-responders*

| Patient | Sex | Age | Number of skin tests positive/3 | Lymphocytes ($10^6$/l) | CYTOTOXICITY ASSAY Mean Corrected % $^{51}Cr$ release DCC | ADCC | PCC | Subpopulation counts: % (absolute count) E | EAC | Null |
|---|---|---|---|---|---|---|---|---|---|---|
| N.F.* | F | 62 | 2 | 472 | 1·1 | 37·7 | 54·5 | 91·0 (430) | 7·6 (36) | 1·4 (7) |
| R.S. | M | 61 | — | 1711 | 5·0 | 20·6 | 35·8 | 73·5 (1258) | 22·1 (378) | 4·4 (75) |
| M.G. | F | 26 | 1 | 1039 | 14·2 | 37·7 | 35·1 | 40·5 (420) | 33·1 (343) | 26·4 (274) |
| J.G. | F | 53 | 1 | 576 | 4·3 | 46·0 | 44·8 | 68·0 (392) | 21·5 (124) | 10·5 (61) |
| A.R. | M | 29 | 2 | 1239 | 8·4 | 45·3 | 57·9 | 63·9 (792) | 14·2 (176) | 21·9 (271) |
| E.C. | F | 53 | 1 | 851 | 11·8 | 33·1 | 40·3 | 84·1 (716) | 22·2 (189) | 0 0 |
| E.T. | M | 54 | 2 | 1176 | 12·6 | 36·1 | 63·6 | 78·1 (919) | 7·8 (92) | 14·1 (166) |
| V.H. | F | 49 | 2 | 1166 | 30·8 | 70·3 | 79·5 | 62·0 (1033) | — — | — — |
| L.B. | F | 72 | 1 | 1222 | 26·0 | 22·9 | 65·7 | 69·5 (849) | — — | — — |
| R.B. | M | 55 | 1 | 1568 | 40·3 | 54·9 | 41·3 | 44·0 (690) | 47·0 (737) | 9·0 (141) |
| J.B. | F | 51 | 0 | 460 | 12·9 | 51·4 | 50·9 | 72·0 (331) | 30·0 (138) | 0 (0) |
| C.M. | M | 29 | 0 | 930 | 1·9 | 14·8 | 22·7 | 70·2 (653) | 34·4 (320) | 0 (0) |
| D.R. | M | 30 | 0 | 762 | 10·1 | 49·4 | 50·3 | 68·6 (523) | 25·7 (196) | 5·7 (43) |
| E.H. | F | 69 | — | 1456 | 0·1 | 21·9 | 2·1 | 52·0 (757) | 34·3 (499) | 13·7 (200) |
| L.S. | M | 59 | 0 | 1904 | 0 | 48·0 | 32·0 | — — | — — | — — |
| N.H. | F | 49 | — | 1802 | 1·9 | 33·8 | 24·5 | 69·9 (1260) | — — | — — |
| N.K.* | F | 58 | — | 4917 | 18·9 | 33·9 | 34·0 | — — | — — | — — |
| E.T. | M | 28 | 1 | 855 | 7·2 | 30·2 | 26·7 | — — | — — | — — |
| J.W. | F | 41 | 0 | 1602 | 6·2 | 10·5 | 21·8 | 65·7 (1053) | — — | — — |
| F.T. | F | 50 | 0 | 2480 | 11·0 | 41·0 | 47·0 | 71·0 (1763) | 26·0 (645) | 2·9 (72) |
| R.G. | M | 22 | 0 | 392 | 15·9 | 48·6 | 61·5 | 66·9 (262) | 29·1 (114) | 4·0 (16) |
| E.S. | F | 55 | 2 | 2060 | 16·6 | 45·1 | 47·2 | 69·9 (1440) | 31·0 (426) | 0 (233) |
| R.M. | M | 38 | 1 | 1564 | 27·9 | 37·8 | 48·7 | 63·2 (988) | 34·7 (543) | 2·1 (33) |
| A.B. | F | 40 | 1 | 2372 | 45·0 | 45·7 | 50·3 | — — | — — | — — |
| J.M. | M | 64 | 3 | 1440 | 5·8 | 14·1 | 32·6 | 46·3 (667) | 43·7 (629) | 10·0 (144) |

* Previous radiation therapy.

— Not tested

TABLE II.—*Test Data for Normal Contols*

| Name | Sex | Age | Lymphocytes ($10^6$/l) | CYTOTOXICITY ASSAY Mean Corrected % $^{51}Cr$ release | | | Subpopulation counts % (absolute count) | | |
|---|---|---|---|---|---|---|---|---|---|
| | | | | DCC | ADCC | PCC | E | EAC | Null |
| D.C. | M | 38 | 1902 | 5·0 | 47·6 | 71·0 | 71·0 (1350) | 19·2 (365) | 9·8 (186) |
| H.B. | M | 32 | 1724 | 9·5 | 44·7 | 67·9 | 75·1 (1294) | 16·3 (281) | 8·6 (148) |
| W.Y. | M | 39 | 2100 | 23·6 | 35·9 | 34·7 | 67·5 (1413) | 23·2 (487) | 9·3 (195) |
| D.B. | F | 39 | 2638 | 27·5 | 52·1 | 33·0 | 71·4 (1884) | 21·9 (577) | 6·7 (177) |
| I.T. | M | 49 | 1692 | 14·2 | 47·2 | 53·1 | 57·8 (978) | 21·3 (360) | 20·9 (354) |
| L.S. | M | 54 | 2090 | 26·3 | 51·3 | 41·4 | 57·1 (1198) | 21·0 (441) | 21·9 (460) |
| S.M. | M | 60 | 1796 | 10·1 | 42·0 | 37·5 | 69·2 (1242) | 24·0 (431) | 6·8 (122) |
| *A.A. | F | 59 | 2200 | 9·7 | 18·4 | 48·6 | 65·9 (1450) | 23·8 (524) | 10·3 (223) |
| M.S. | M | 33 | 2689 | 20·8 | 38·6 | 9·8 | 75·9 (2041) | 13·7 (368) | 10·4 (280) |
| G.T. | M | 66 | 1012 | 15·1 | 23·8 | 58·6 | 74·9 (758) | 11·8 (119) | 13·3 (134) |
| *J.B. | M | 63 | 2138 | 22·3 | 38·0 | 44·1 | 70·7 (1512) | 25·6 (547) | 3·7 (75) |
| *B.W. | M | 51 | 1609 | 26·9 | 40·3 | 40·6 | 58·4 (939) | 16·6 (267) | 25·0 (402) |
| *P.W. | M | 61 | 2099 | 19·0 | 30·8 | 47·2 | 53·0 (1113) | 26·3 (552) | 20·7 (435) |
| C.G. | M | 33 | 1804 | 16·2 | 39·3 | 43·6 | 63·5 (1145) | 33·0 (595) | 3·5 (63) |
| A.D. | M | 53 | 1878 | 21·3 | 55·0 | 37·3 | 75·9 (1425) | 13·1 (246) | 11·0 (207) |
| S.F. | F | 54 | 2094 | 13·7 | 35·1 | 27·7 | 79·3 (1659) | 20·9 (435) | 0 (0) |
| *D.D. | M | 58 | 2601 | 8·0 | 34·0 | 45·7 | 63·5 (1651) | 33·5 (571) | 3·0 (78) |
| *R.N. | M | 68 | 1880 | 19·7 | 45·9 | 43·1 | 70·0 (1316) | 25·6 (481) | 4·4 (83) |
| A.L. | F | 47 | 1524 | 6·1 | 39·1 | 57·1 | 58·2 (887) | 16·3 (248) | 25·5 (389) |
| S.S. | M | 42 | 1999 | 2·0 | 46·8 | 29·2 | 53·0 (1060) | 26·5 (530) | 20·5 (410) |
| *R.H. | M | 49 | 2198 | 15·0 | 45·2 | 45·1 | 67·2 (1477) | 28·0 (615) | 4·8 (100) |

* Patient's relatives

U.S.A.), and *Candida albicans* antigen 0·33% (Bencard, Brentford, Middlesex, U.K.) were used as the skin-test agents. The tests were performed before treatment, using an intradermal inoculation of 0·1 ml into the forearm. Results of the skin tests were read at 48 h and the diameter of the induration and erythema measured. A positive reaction was defined as 5 mm or more of induration at 48 h.

*Lymphocyte preparations.*— Lymphocyte suspensions for cytotoxic assays were prepared from defibrinated peripheral blood after incubation with finely divided iron and sedimentation in 1% methylcellulose at 37°C using a magnet. The lymphocyte-rich supernatant was washed × 3 in minimal essential medium (MEM) and the concentration adjusted to $3 \times 10^5$ cell/ml in MEM supplemented with 10% heat-inactivated foetal calf serum, 2mM glutamine, 100 iu/ml streptomycin, 200 iu/ml penicillin and $NaHCO_3$ buffer. The lymphocytes comprised 96% or more of the leucocytes present in the suspensions. Lymphocyte preparations for the rosette tests were obtained by Ficoll-Triosil gradient centrifugation. After incubation with finely divided iron, heparinised blood was diluted with an equal volume of phosphate-buffered saline (PBS) and layered on a mixture of Triosil and 9% Ficoll. After centrifugation, interface cells were collected and the cells washed × 3 with PBS. The lymphocyte purity with this method of separation was 93% or more of the leucocytes counted, the remainder comprising 5% monocytes and 2% neutrophils. Differential counts were performed on smear preparations after Jenner-Giemsa staining.

*E rosettes.*— Washed sheep red blood cells (SRBC, Wellcome Reagents Ltd) were diluted to a concentration of $2 \times 10^8$/ml in foetal calf serum (FCS) and 0·2 ml of this suspension was mixed with $10^6$ lymphocytes (in 0·2 ml FCS). The cells were sedimented by gentle centrifugation and incubated for 18 h at 4°C. The cells were then gently resuspended and examined immediately in a haemocytometer, the number of cells binding 3 or more SRBC was determined as a percentage of the total lymphocytes. All lymphocyte preparations were tested in triplicate and the means calculated.

*EAC rosettes.*—A suspension of washed SRBC (containing 0·2 ml packed-cell volume) was incubated for 30 min at 37°C with 4 ml of rabbit anti-SRBC serum (1/500 final dilution). The antibody-labelled SRBC were then washed × 3 and incubated for a further 30 min at 37°C, with human serum as a complement source (1/20 final dilution). The labelled SRBC were again washed × 3 and the concentration adjusted to $2 \times 10^8$/ml in PBS. Lymphocytes ($10^6$ in 0·2 ml PBS) were incubated with 0·2 ml of labelled SRBC for 30 min at 37°C with shaking, and then examined in a haemocytometer to determine the percentage of rosette-forming cells.

Null-cell percentage was determined from (100-sum (E + EAC rosette percentages)).

*Cytotoxic assay.*—Chang cells labelled with $^{51}Cr$ sodium chromate (sp. act. 100–350 mCi/mmol, Amersham, Bucks.) were washed and diluted to give a final concentration of $10^4$ cell/ml in supplemented MEM. The $^{51}Cr$-release assay of non-specific cytotoxicity against antibody-sensitised Chang target cells (MacLennan and Loewi, 1968; Perlmann and Holm, 1968) and the PHA-stimulated lymphocytotoxicity against labelled Chang cells (Holm *et al.*, 1964; Holm and Perlmann, 1967) were performed as follows. The cultures were set up in triplicate, containing 1 ml of lymphocytes and 1 ml of Chang cells. The test was divided into 3 sections. Chang cells were tested with lymphocytes alone (DCC) with lymphocytes and rabbit anti-Chang serum diluted 1:$10^5$ (ADCC) and lymphocytes with PHA (Purified Phytohaemagglutinin, Wellcome Reagents Ltd) 3 μg/ml (PCC). Control tubes were set up with each experiment, containing Chang cells alone, with antibody or with PHA, but without lymphocytes. These tubes gave the spontaneous $^{51}Cr$ release. Maximal $^{51}Cr$ release was obtained by lysis with distilled water. After standing at 37°C in a 5% $CO_2$ incubator for 20 h, the tubes were centrifuged and 0·5 ml supernatant transferred to empty tubes and counted on a gamma counter. The ct/min was punched on to a paper tape which was processed by a digital computer.

The $^{51}Cr$ % release was computed for each tube from

$$\frac{\text{Supernatant ct/min} \times 4}{\text{Total ct/min (pellet + supernatant)}} \times 100$$

The maximal release was 88–99% and the spontaneous release was 18–31%.

Results were expressed as the mean corrected % $^{51}Cr$ release (MCR) of the triplicates, and obtained from

$$\frac{\text{Experimental } ^{51}\text{Cr \% release} - \text{spontaneous } ^{51}\text{Cr \% release}}{\text{Maximal } ^{51}\text{Cr \% release} - \text{spontaneous } ^{51}\text{Cr \% release}} \times 100$$

The s.d. between triplicates was 3%

RESULTS

The two patient groups, responders and non-responders, and the normal control group were compared for differences in lymphocyte count, DCC, ADCC, PCC, E rosette %, EAC rosette %, "null" cell % and the absolute E, EAC and null-cell counts. Cytotoxic capacity (% MCR × lymphocyte count) was also calculated for DCC, ADCC and PCC for individual subjects and the median values compared. Non-parametric statistical techniques have been used because of non-normality of data. As there were 3 groups, the Kruskal-Wallis "one-factor analysis of variance for ranks" was used initially and, if statistically significant, each pair of groups was then contrasted using the Mann-Whitney U test at a reduced level of statistical significance (1%).

TABLE III.—*% Metastatic Involvement in Responders and Non-responder Patient Groups*

| | Responders | Non-responders |
|---|---|---|
| Pulmonary/Hepatic | 38 | 40 |
| Osseous/Cerebral | 8 | 8 |
| Nodal | 100 | 76 |
| Cutaneous | 70 | 80 |

The distribution of metastases was similar in the responder and non-responder patient groups (Table III). There was no other obvious clinical difference between these two groups before systemic therapy commenced.

The lymphocyte count, cytotoxic capacity for DCC and ADCC, E rosette and "null" cell counts were found to be significantly different between the 3 groups using the Kruskal-Wallis test (Table IV). The additional analysis demonstrated that the difference was statistically significant between the *non-responder* and control groups, with the following *P* values: lymphocyte count 0·001, DCC

TABLE IV.—*Median Values of* In vitro *Lymphoid Tests for Patients and Normals (range in brackets)*

| | Non-responders | Responders | Normals | *P* |
|---|---|---|---|---|
| Lymphocytes/μl | 1440 (392–4917) | 1536 (305–2530) | 1999 (1012–2689) | 0·004* |
| DCC % MCR | 11·0 (0–45·0) | 18·1 (0·67·2) | 15·1 (2·0–27·5) | 0·13 |
| ADCC % MCR | 37·7 (10·5–70·3) | 55·1 (5·3–90·3) | 40·3 (18·4–55·0) | 0·11 |
| PCC % MCR | 44·8 (2·1–79·5) | 57·1 (16·9–73·6) | 43·6 (9·8–71·0) | 0·27 |
| DCC Capacity | 66 (1–712) | 179 (0–751) | 220 (27–1387) | 0·021* |
| ADCC Capacity | 251 (92–1111) | 566 (16–1090) | 520 (159–903) | 0·021* |
| PCC Capacity | 313 (20–1115) | 570 (50–1072) | 523 (176–792) | 0·061 |
| E Rosette % | 68·6 (40·5–91·0) | 66·3 (39·3–79·1) | 67·5 (53·0–79·3) | 0·78 |
| EAC Rosette % | 29·1 (7·6–47·0) | 23·9 (20·5–45·7) | 21·9 (11·8–33·5) | 0·11 |
| Null % | 4·4 (0–26·4) | 10·2 (0–27·5) | 9·8 (0–25·5) | 0·24 |
| E Rosette/μl | 716 (262–1763) | 1036 (176–2001) | 1294 (758–2041) | 0·002* |
| EAC Rosette/μl | 320 (36–737) | 401 (109–998) | 441 (119–615) | 0·24 |
| Null cells/μl | 72 (0–274) | 128 (0–223) | 186 (0–460) | 0·01* |

* Statistically significant, ($P < 0·05$) Kruskal-Wallis one-factor analysis of variance for ranks.

capacity 0·006, ADCC capacity 0·004, E rosette count 0·0002, and "null" cell count 0·004.

Analysis of the other test values revealed that the differences between the groups were *not* statistically significant, although examination of median values showed a consistent pattern between the 3 groups (Table IV).

The median values of the *responder* group: lymphocyte count 1536; DCC 18·1; ADCC 55·1; PCC 57·1 and their cytotoxic capacity (179, 566, 570 respectively), were all higher than in the non-responder group, 1440, 11·0, 37·7, 44·8 and 66, 251, 313 respectively.

Higher values for null cell %, E-rosette counts, EAC-rosette counts and null-cell counts were also found in the responder group (10·2, 1036, 401, 128 respectively) than in the non-responder group (4·4, 716, 320, 72 respectively). The E, EAC rosettes and null counts were highest in normals, with respective values: 1294, 441, 186.

The values of E rosette % for the non-responders (68·6), responders (66·3) and normals (67·5) showed very little difference. The EAC rosette %, however, was higher in non-responders (29·1) than in responders (23·9) and controls (21·9). The skin reactivity to "recall" antigens was similar in both patient groups. The responder group showed 10/33 positive

FIG. 1.—Sequential reaction of lymphocyte toxicity (see text for details) of melanoma patients not responding to 4 or more courses of chemo-immunotherapy (CT + BCG).

FIG. 2.—As for Fig. 1, for 3 melanoma patients responding to chemotherapy and one untreated.

reactions (30·3%) compared with 21/63 (33·3%) in the non-responding group.

The sequential study of lymphocyte cytotoxicity included 5 patients who were non-responders (Fig. 1), 3 who were responders and one untreated patient with "stable" disease (Fig. 2). The untreated patient, D.M. showed much less variation in sequential tests than the patients undergoing chemo-immunotherapy.

Comparison of sequential cytotoxicity values for the individual patients showed no significant changes. The patients' results were also pooled for similar occasions, but again no significant difference was found between the cytotoxicity values, using a Friedman 2-way non-parametrical analysis.

### DISCUSSION

This study demonstrates differences in lymphoid-cell function between melanoma patients and healthy controls. It also suggests a difference in pre-treatment lymphoid-cell function between patients who subsequently respond and those who fail to respond to systemic therapy. The 2 patient groups ("responder", "non-responder") at the time of immunological investigation were clinically similar, particularly with regard to the distribution of metastases (Table III). It is, therefore, unlikely that clinical response or the immunological profile were dictated by a particular pattern of metastatic desease. However, this does not necessarily imply that the pre-treatment tumour burden was the same in both patient groups. It might be expected that if the "responders" had a smaller tumour mass than the "non-responders", they would be more likely to respond to therapy. Patient tumour mass is as yet impossible to quantitate precisely, but in the present investigation, there was no obvious difference between the two groups.

The lymphocyte count was significantly less in the non-responder patient group than in the healthy control group. The median lymphocyte count for the responder group was also lower than that of the control group, but higher than that of the non-responder group; the differences between patient groups were not however significant. This relationship between response and lymphocyte count has not previously been clearly defined, although lymphopenia has, in some series, been related to poor survival (Riesco, 1970; Papatestas and Kark, 1974).

The null-cell and E-rosette counts demonstrated a similar significant difference between non-responder patients and healthy controls, with the median values of the responder group lying midway between the other two. There have been previous reports of T-cell depression in cancer patients' blood (Wybran and Fudenberg, 1973) but EAC resetting cells in a variety of solid tumours, including melanoma, have had normal values (Anthony *et al.*, 1975; Peter *et al.*, 1975; Whitehead *et al.*, 1976). The presence of null cells in the present study was associated with lack of response. The increase in null-cell number is partly a reflection of the reduction in E- and EAC-rosette numbers, and it is not possible to determine whether the lack of response was associated with reduced E-rosette levels alone or whether the change in null cells was also important. A similar situation was observed in patients with bronchial carcinoma, although in this study it was percentage of cells and not absolute number that demonstrated significant differences (Anthony *et al.*, 1975). This is in contrast to the present investigation, where E rosette and EAC rosette percentages showed very little difference between patient groups and normal controls. It is perhaps reasonable to analyse both percentages and absolute numbers until more data have accumulated, to allow the emphasis to be placed upon either one of the values.

Delayed skin hypersensitivity to antigens has been used as an *in vivo* measure of cell-mediated immunity in melanoma patients. In the present study, reactivity in both patient groups was depressed to about the same extent and no significant difference in skin reaction could be found between responders and non-responders. These data support other work describing depressed skin reactivity in melanoma (Catalona, Sample and Chretien, 1973) but is at variance with reports of normal skin reactivity (Ziegler *et al.*, 1969) and of the clinical significance attached to such tests (Morton *et al.*, 1970). However, the use of "skin test reactivity" should be encouraged, despite the present and other reports that lack statistically significant differences, as it is the only *in vivo* method routinely available for immunological evaluation of patients.

Immune reactivity *in vitro* has been related to tumour stage in some studies; "tumour-specific "cellular immunity being greater in localised than in generalised disease (Cochran *et al.*, 1973) and a reduction in immunity being associated with advancing disease (de Vries, Rumke and Bernheim 1972; Heppner *et al.*, 1973). However, other authors, using PHA-induced lymphocyte blastogenesis have found either no difference between patients and controls (Ziegler *et al.*, 1969; Catalona *et al.*, 1973) or a depression in melanoma patients unrelated to the clinical situation (Lui *et al.*, 1975; Gatti, Garrioch and Good, 1970). Lymphocyte microcytotoxicity assays have also failed to relate to response rates (Vanwijck, Bouillenne and Malek-Mansour, 1975; Catalona *et al.*, 1973; Berkelhammer *et al.*, 1975). Little attention has been directed at "K"-cell activity as measured by ADCC, but lower reactivity in cancer patients than in normal controls has been noted (Ting and Terasaki, 1974; Peter *et al.*, 1975). However, the relationship with response to therapy was not commented upon.

In the present study, a consistent pattern of cytotoxicity was seen in the 3 groups. The medians for the responder patients showed no immunodepression relative to controls. However, "non-T-cell" (DCC, ADCC) cytotoxicity was depressed in the non-responders.

But, this depression was only statistically significant if "non-T-cell" function was expressed in terms of the lymphocyte count. The calculation is analagous to that used in generating the rosetting-cell counts. The implication is also similar—that the "total" cytotoxicity of a functionally defined subpopulation might be a more biologically meaningful number to use in statistical analysis.

The sequential study of cytotoxicity in 8 patients failed to demonstrate any consistent pattern with repeated courses of chemotherapy and BCG vaccination. However, these patients, both responders and non-responders, did exhibit more fluctuation in cytotoxicity than the untreated patient with "stable" disease (Figs. 1, 2) or normal controls assayed on several occasions (Thatcher *et al.*, 1977) suggesting perturbation of cytotoxicity by the chemo-immunotherapy and/or by alteration in tumour load. A similar lack of clinical correlation with a microcytoxicity assay has been noted in melanoma patients receiving immunotherapy alone (Berkelhammer *et al.*, 1975).

This investigation suggests that a high level of "non-T-cell" cytotoxicity might be important for a successful therapeutic outcome in metastatic melanoma. The median values of the DCC and ADCC assays (albeit only significantly different for "DCC capacity") were greater in responders, than in non-responders, and might be a reflection of the higher concentrations of non-E-rosetting cells circulating in these patients. Although T-cell numbers and PCC were higher in responders than in non-responders, the level of PCC was near normal in the latter group and, furthermore, these differences in PCC were not statistically significant. A relationship between "non-T-cell" cytotoxicity and clinical response might therefore be postulated.

### REFERENCES

ANTHONY, H. M., KIRK, J. A., MADSEN, K. E., MASON, J. K. & TEMPLEMAN, G. H. (1975) E and EAC Rosetting Lymphocytes in Patients with Carcinoma of the Bronchus. I. Some Parameters of the Test and of its Prognostic Significance. *Clin. exp. Immunol.*, **20**, 29.

BERKELHAMMER, J., MASTRANGELO, M. J., LAUCIUS, J. F., BODURTHA, A. J. & PREHN, R. T. (1975) Sequential *In vitro* Reactivity of Lymphocytes from Melanoma Patients Receiving Immunotherapy Compared with the Reactivity of Lymphocytes from Healthy Donors. *Int. J. Cancer*, **16**, 571.

BIANCO, C., PATRICK, R. & NUSSENZWEIG, V. (1970) A Population of Lymphocytes Bearing Membrane Receptor for Antigen-antibody Complement Complexes. *J. exp. Med.*, **132**, 702.

CATALONA, W. J., SAMPLE, W. F. & CHRETIEN, P. P. (1973) Lymphocyte Reactivity in Cancer Patients Correlation with Tumour Histology and Clinical Stage. *Cancer, N. Y.*, 31. 65.

COCHRAN, A. J., MACKIE, R. N., THOMAS, C. E., GRANT, R. N., CAMERON-MOWAT, D. E. & SPILG, W. G. S. (1973) Cellular Immunity to Breast Carcinoma and Malignant Melanoma. *Br. J. Cancer* **28**, Suppl., 1, 153.

DE VRIES, J. E., RUMKE, P. & BERNHEIM, J. L. (1972) Cytotoxic Lymphocytes in Melanoma Patients. *Int. J. Cancer*, **9**, 567.

DE VRIES, J. E., CORNIAN, S. & RUNKS, P. (1974) Cytotoxicity of Non-T *versus* T Lymphocytes from Melanoma Patients and Healthy Donors on Short and Long Term Cultured Melanoma Cells. *Int. J. Cancer*, **14**, 427.

FOSSATI, G., COLNAGHI, M. I., DELLA PORTA, G., CASCINELLI, N. & VERONESI, U. (1971) Cellular and Humoral Immunity against Human Malignant Melanoma. *Int. J. Cancer*, **8**, 344.

GATTI, R. A., GARRIOCH, D. B. & GOOD, R. A. (1970) Depressed PHA Responses in Patients with Non-lymphoid Malignancies. *Proceedings of the* Fifth Leukocyte Culture Conference. New York: Academic Press, p. 339.

HARDING, B., PUDIFIN, D. J., GOTCH, F. M. & MACLENNAN, I. C. M. (1971) Cytotoxic Lymphocytes from Rats Depleted of Thymus Processed Cells. *Nature, New Biol.*, **232**, 80.

HEPPNER, G. H., STOLBACH, L., BYRNE, M., CUMMINGS, F. J., McDONOUGH, E. & CALABRESI, P. (1973) Cell Mediated and Serum Blocking Reactivity to Tumour and Antigens in Patients with Malignant Melanoma. *Int. J. Cancer*, **11**, 245.

HERSEY, P., EDWARDS, A., EDWARDS, J., ADAMS, E., MILTON, G. W. & NELSON, D. S. (1975) Specificity of Cell-mediated Cytotoxicity against Human Melanoma Lines: Evidence for Non-specific Killing by Activated T Cells. *Int. J. Cancer*, **16**, 173.

HOLM, G., PERLMANN, P. & WERNER, B. (1964) Phytohaemagglutinin-induced Cytotoxic Action of Normal Lymphoid Cells and Cells in Tissue Culture. *Nature, Lond.*, **203**, 841.

HOLM, G. & PERLMANN, P. (1967) Quantitative Studies on Phytohaemagglutinin Induced Cytotoxicity by Human Lymphocytes against Homologous Cells in Tissue Culture. *Immunology*, **12**, 525.

JONDAL, M., HOLM, G. & WIGZELL, H. (1972) Surface Markers on Human T and B Lymphocytes. I. A Large Population of Lymphocytes Forming Non-immune Rosettes with Sheep Red Blood Cells. *J. exp. Med.*, **136**, 207.

JONDAL, M. & PROSS, H. (1975). Surface Markers on Human B and T Lymphocytes. VI. Cytotoxicity against Cell Lines as a Functional Marker for Lymphocyte Sub-populations. *Int. J. Cancer*, **15**, 596.

LAY, W. H., MENDES, N. F., BIANCO, C. & NUSSENZWEIG, V. (1971) Binding of Sheep Red Blood Cells to a Large Population of Human Lymphocytes. *Nature, Lond.*, **230**, 531.

LEWIS, M. G., IKONOPISOV, R. L., NAIRN, R. C., PHILLIPS, T. M., HAMILTON-FAIRLEY, G., BODENHAM, D. C. & ALEXANDER, P. (1969) Tumour Specific Antibodies in Human Malignant Melanoma and their Relationship to Extent of Disease. *Br. med J.*, iii. 547.

LUI, V. K., KARPUCHAS, J., DENT, P. B., McCULLOCH, P. B. & BLAJCHMAN, M. A. (1975) Cellular Immunocompetence in Melanoma Effect of Extent of Disease and Immunotherapy. *Br. J. Cancer*, **32**, 323.

MACLENNAN, I. C. M. & LOEWI, G. (1968) The Effect of Specific Antibody to Target Cells on their Specific and Non-specific Interaction with Lymphocytes. *Nature, Lond.*, **219**, 1069.

MACLENNAN, I. C. M. (1972) Antibody in the Induction and Inhibition of Lymphocyte Cytotoxicity. *Transplantn. Rev.*, **13**, 67.

MORTON, D. L., EILBER, F. R., MALMGREN, R. A. & WOOD, D. C. (1970) Immunological Factors which Influence Response to Immunotherapy in Malignant Melanoma. *Surgery*, **68**, 158.

O'TOOLE, C., STEJSKAL, V., PERLMANN, P. & KARLSSON, M. (1974) Lymphoid Cells Mediating Tumour Specific Cytotoxicity to Carcinoma of the Urinary Bladder; Separation of the Effector Population Using a Surface Marker. *J. exp. Med.*, **139**, 457.

PAPATESTAS, A. E. & KARK, A. E. (1974) Peripheral Lymphocyte Counts in Breast Carcinoma. *Cancer, N.Y.*, **34**, 2014.

PAVIE-FISCHER, J., KOURILSKY, F. M., PICARD, F., BAUZET, P. & PUISSANT, A. (1975) Cytotoxicity of Lymphocytes from Healthy Subjects and from Melanoma Patients against Cultured Melanoma Cells. *Clin exp. Immunol.*, **21**, 430.

PERLMANN, P. & HOLM, G. (1968) Studies on the Mechanisms of Lymphocyte Cytotoxicity. In *Mechanisms of Inflammation Induced by Immune Reactivity*. Eds P. A. Miescher & P' Graber. Basle: Schaber & Co., p. 325.

PERLMANN, P. & HOLM, G. (1969) Cytotoxic Effects of Lymphoid Cells *In vitro*. *Adv. Immun.*, **11**, 117.

PETER, H. H., PAVIE-FISCHER, J., FRIDMAN, W. H., AUBERT, C., CESARINI, J. P., ROUBIN, R. & KOURILSKY, F. M. (1975) Cell-mediated Cytotoxicity *In vitro* of Human Lymphocytes against a Tissue Culture Melanoma Cell Line (IGR3). *J. Immun.*, **115**, 539.

PETER, H. H., KALDEN, J. R., SEELAND, P., DIEHL, V. & ECKERT, G. (1975) Humoral and Cellular Immune Reactions "In vitro" against Allogeneic and Autologous Human Melanoma Cells. *Clin. exp. Immun.*, **20**, 193.

RIESCO, A. (1970) Five-year Cancer Cure: Relation to Total Amount of Peripheral Lymphocytes and Neutrophils. *Cancer*, **25**, 135.

TAKASUGI, M., MICKEY, M. R. & TERASAKI, P. I. (1973) Reactivity of Lymphocytes from Normal Persons, on Cultured Tumour Cells. *Cancer Res.*, **33**, 2898.

THATCHER, N., GASIUNAS, N., POTTER, M. R., CROWTHER, D. & MOORE, M. (1977) Effects of

Intermittent 5 Fluorouracil and Adriamycin on various Immune Parameters in Carcinoma Patients with Reference to the Tumor load. *Cancer Immun. Immunother.*, (In press).
TING, A. & TERASAKI, P. I. (1974) Depressed Lymphocyte Mediated Killing of Sensitised Targets in Cancer Patients. *Cancer Res.*, **34**, 2694.
VANWIJCK, R., BOUILLENNE, C. & MALEK-MANSOUR, S. (1975) Potentiation and Arming of Lymphocyte Mediated Immunity by Sera from Melanoma Patients. *Eur. J. Cancer.* **11**, 267.
WHITEHEAD, R. H., THATCHER, J., TEASDALE, C., ROBERTS, G. P. & HUGHES, L. E. (1976) T. and B Lymphocytes in Breast Cancer. Stage Relationship and Abrogation of T Lymphocyte Depression by Enzyme Treatment *In vitro*. *Lancet*, i, 330.
WYBRAN, J. & FUDENBERG, H. H. (1973) Thymus Derived Rosette Forming Cells in Various Human Disease States: Cancer, Lymphoma, Bacterial and Virus Infections and other Diseases. *J. clin. Invest.*, **52**, 1026.
ZIEGLER, J. L., LEWIS, M. G., LUYOMBYA, J. M. S. & KIRYABWIE, J. W. M. (1969) Immunologic Studies in Patients with Malignant Melanoma in Uganda. *Br. J. Cancer*, **23**, 729.